# Cloning and Functional Characterization of a Novel *β-GRP* Gene From *Melanotus cribricollis*

**DOI:** 10.1093/jisesa/ieac051

**Published:** 2022-09-09

**Authors:** Bihuan Ye, Qiyan Song, Haibo Li, Jianjun Shen, Chenyou Wu, Jinping Shu, Yabo Zhang

**Affiliations:** Zhejiang Academy of Forestry, Hangzhou 310023, China; Zhejiang Academy of Forestry, Hangzhou 310023, China; Zhejiang Academy of Forestry, Hangzhou 310023, China; Zhejiang Academy of Forestry, Hangzhou 310023, China; Zhejiang Academy of Forestry, Hangzhou 310023, China; Research Institute of Subtropical Forestry, Chinese Academy of Forestry, Hangzhou 311400, China; Research Institute of Subtropical Forestry, Chinese Academy of Forestry, Hangzhou 311400, China

**Keywords:** *Melanotus cribricollis*, *β-GRP*, anti-fungal, immune regulation

## Abstract

In this study, a novel *β*-1,3-glucan recognition protein gene (*β-GRP*) was identified from *Melanotus cribricollis*, and its potential role in the immune response was investigated. The full length of *β-GRP* cDNA (Accession Number: MT941530) was 1644 bp, encoding a protein composed of 428 amino acids. The theoretical molecular weight and the isoelectric point were 51.53 kDa and 6.17, respectively. The amino acid sequence of *β*-GRP from *M. cribricollis* was closely related to that of*. β-*GRP-like from *Photinus pyralis*, and was predicted to contain conserved GH16 domain without glucanase active site. The results of real-time quantitative PCR showed that fungal infection of *Metarhizium pingshaense* may significantly upregulated the expression level of *β-GRP* gene. The RNAi suppression of *β-GRP* gene expression significantly increased the corrected cumulative mortality. Meanwhile, antimicrobial peptide genes *defensin* and *lysozyme* were significantly downregulated after interference of *β-GRP.* Taken together, these results suggest that *β-GRP* of *M. cribricollis* probably participates in the host immune system by mediating the expression of antimicrobial peptides. This study provides comprehensive insights into the innate immune system of insect larvae.

## Graphical Abstract

**Graphical Abstract:**
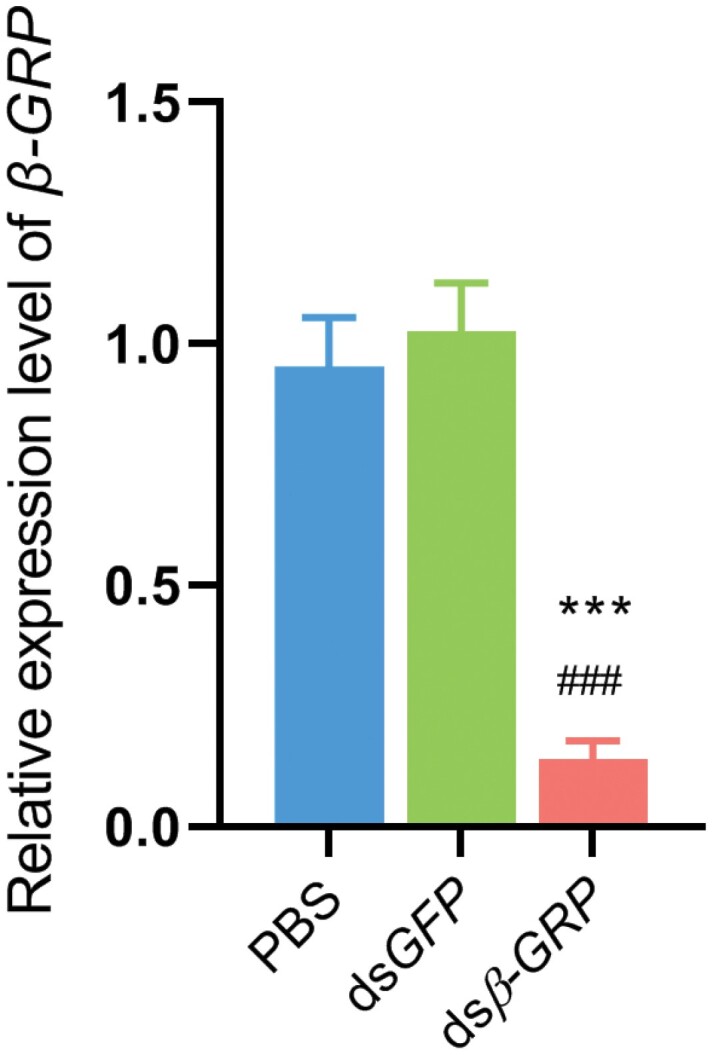

The innate immune system is the basic defense system of insects against pathogen invasion because they lack adaptive immunity (Ochiai and Ashida 2000). In response to pathogen attack, the pattern recognition receptors (PRRs) of insects bind with pathogen-associated molecular patterns (PAMPs) and then activate downstream pathways, mainly involves phenoloxidase (proPO) cascades, Toll and Imd signaling pathway, which further promote the melanization cascade or induce the production of antimicrobial peptides (AMPs) (Hoffmann 2003, Mishima et al. 2009, Matskevich et al. 2010). Insect PRRs include proteins, such as *β*-1,3-glucan recognition protein (*β*-1,3-GRP), Gram-negative bacteria-binding protein (GNBP), and peptidoglycan recognition protein (PGRP), among which *β*-1,3-GRP and GNBP are classified into a subfamily of PRR because of their high homology (Royet 2004, Vogel et al. 2014). β-1,3-GRP was first thought as component of the proPO cascade in silkworm (Ochiai and Ashida 1988). A previous study reported that *β*-1,3-GRP/GNBP3 is a key PRR with strong affinity with *β*-1,3-glucan, a cell wall component of fungi. Upon binding to fungi, it evokes the Toll and proPO pathways in invertebrates to defend against fungal invasion (Takahasi et al. 2009). Insect βGRP homologs consist of a highly conserved N-terminal domain and a β-1,3-glucanase-like domain in the C-terminal region (Ochiai and Ashida 2000, Hoffmann 2003). Accordingly, *β-1,3-*GRP is an important component of insects to resist foreign invaders (Lemaitre and Hoffmann 2007, Dai et al. 2013). To date, the function of the *β*-1,3-GRP homolog has been well described in different insects but remains elusive in *Melanotus cribricollis* (Faldermann) (Coleoptera: Elateridae) larvae. 

Larvae of *M. cribricollis* are the dominant species of bamboo shoot wireworms, which are important shoot-stage pests in bamboo forests. The damage rate of bamboo forests can be as high as 80%. The number of insects feeding on a fresh bamboo shoot can reach nearly 20. *M. cribricollis* lives underground and has a long history, making it difficult to prevent and control (Shu et al. 2012). Previously, the control of bamboo shoot wireworms in bamboo forests relied on chemical agents, such as fipronil, chlorpyrifos, imidacloprid, and phoxim (Song et al. 2009). However, chemical agents such as chlorpyrifos have been gradually banned because of the increasingly prominent problems of pesticide residues, environmental pollution, and drug resistance. Biocontrol bacteria, such as *Metarhizium pingshaense* Q.T. Chen & H.L. Guo (Ascomycota: Hypocreales), have the advantages of environmental protection and sustainability, and they have been widely studied and applied in recent years. *M. pingshaense* has pathogenic effects on more than 200 pests, including those belonging to the orders Coleoptera, Lepidoptera, Orthoptera, Diptera, and Homoptera, and is a biocontrol strain with great potential (Roberts and St Leger 2004).

In our previous study, we identified using next-generation sequencing (RNA-seq) that *β-GRP/GNBP* genes are significantly upregulated after infection with *M. pingshaense* WP08 strain for 7 d (Ye et al. 2018). This study suggests that *β-GRP* of *M. cribricollis* was probably involved in immune regulation. The current research on insect immune mechanism focused on the silkworm and fruit flies, and few reports elucidated the immune mechanism of underground phytophagous pests. Identifying the immune-related genes of *M. cribricollis* in bamboo forests will provide not only reference information for immune mechanism research in other insect but also provide target genes for transgenic insect resistance technology and lay a foundation for the development of high-efficiency biocontrol strains that inhibit the insect immune system.

## Materials and Methods

### Animals and Pathogens

Healthy larvae of *M. cribricollis* with the same body weight were collected from the bamboo forest of Zaoyuan in Deqing County, Zhejiang Province, China and fed with fresh bamboo shoots indoors. *M. pingshaense* WP08 strain was provided by the Forest Protection Research Group of the Institute of Subtropical Forestry, Chinese Academy of Forestry. It was inoculated on potato dextrose agar and cultured at 25°C for approximately 3–4 wk to collect conidia for use. The test soil was collected from the early bamboo garden in Hangzhou, sieved through a 40-mesh screen, sterilized at high temperature (121°C) for 1 h, and then dried. A *M. pingshaense* WP08 conidia suspension was prepared using 0.1% Tween 80 and then mixed with the sterilized test soil for use. The final concentration of conidia was adjusted to 1.2 × 10^7^ pcs·g^−1^ soil. Humidity was adjusted to 10% ± 1% (TZS Soil Moisture Quick Tester, Zhejiang Tuopu Instrument Co., Ltd.).

## Cloning of Full-length *β*-*GRP*

### Total RNA Isolation, cDNA Synthesis, and Gene Cloning

The middle part of the *M. cribricollis* larval body was cut with scissors, placed in a tube, and then quickly placed in liquid nitrogen to cool down to prevent RNA degradation. Total RNA was extracted using TRIzol reagent (Takara, Japan). RNA integrity, concentration, and purity were detected using RNA electrophoresis and a NanoDrop 2000 spectrophotometer (Thermo, USA).

cDNA first-strand synthesis was performed using the PrimeScript Reagent kit with gDNA Eraser (Takara, Japan) in accordance with the manufacturer’s instructions. The primer pair (*Mcβ-GRP*-734F/*Mcβ-GRP*-1152R) was designed according to the Unigene C54949.Graph_c0 sequence acquired by the transcriptomic data of *M. cribricollis* in our previous study (Ye et al. 2018) and used to amplify the intermediate fragment of *β*-*GRP* (Table 1). The 3ʹ RACE cDNA and 5ʹ RACE cDNA of the targeted β-*GRP* gene were synthesized using the 3ʹ RACE kit (Bioteke, China) and SMARTer RACE kit (Takara, Japan), respectively. The specific primers are shown in Table 1. The RACE-PCR program was performed following the manufacturer’s protocol. The PCR products were purified with the Gel Recovery Kit (TSINGKE, China) in accordance with the manufacturer’s protocol. Obtained *β*-*GRP* DNA fragments were further sequenced using Tsingke Biotechnology Co., Ltd. (Beijing, China).

**Table 1.** Summary of primers used in this study

Primers	Purpose	Sequences (5ʹ−3ʹ)
	**RACE**	
*Mcβ-GRP*-734F	PCR of intermediate fragment 1st PCR of 3ʹ RACE	AATCAGCCAGAATAACGTCAC
*Mcβ-GRP*-1152R	PCR of intermediate fragment	CCTCAGGAGAAGTCCACGAA
*Mcβ-GRP*-3ʹ RACE Inner1	2nd PCR of 3ʹ RACE	AACGACGACGATGAGAAT
5ʹ GSP	1st PCR of 5ʹ RACE	GATTACGCCAAGCTTATCAACTGTTCCGATCTCATGCGAGTCT
5ʹ NGSP	2nd PCR of 5ʹ RACE	GATTACGCCAAGCTTTGGAGCCCAAATCAAGCTGAACACAT
	**Real-time PCR analysis**	
*Mcβ-GRP-*qF	qRT-PCR of *β-GRP*	CCCCAGACATTCCTCGTATTG
*Mcβ-GRP-*qR		ATCACTTGGAGTTGGAGTTGG
Def c44149.graph_c0/F1	qRT-PCR of *defensin*	TGGAAAGCGATAGTGAAGGTG
Def c44149.graph_c0/R1		GTGATTGACAGCAAACTTGGG
Lys c48579_graph_c0/F	qRT-PCR of *lysozyme*	GTATTTGTCAAGCTGCGTCATC
Lys c48579_graph_c0/R		CGTTGGCACAAATCTGAAACTG
PGRP c22434.graph_c0/F1	qRT-PCR of* PGRPs*	TGTCGTACTTCTGGCTATCATTG
PGRP c22434.graph_c0/R1		TGTGATGGAGGGTTTACTTGC
RPS27a-qF	Reference gene	CTTGTCCTGAATCTTTGCCTTG
RPS27a-qR		GTTCTTTTGGTAGCGTGTCATG
RPS3-qF	Reference gene	CAATAGCGCACAAACCACG
RPS3-qR		TGTATTGGGTGAAAAGGGAAGG
	**Double-strand RNA synthesis**	
*GFP*-F	dsRNA synthesis of *GFP*	ATGGTGAGCAAGGGCGAGGAG
*GFP*-R		TTACTTGTACAGCTCGTCCATGCC
T7-*GFP*-F		ggatcctaatacgactcactataggATGGTGAGCAAGGGCGAGGAG
T7-*GFP*-R		ggatcctaatacgactcactataggTTACTTGTACAGCTCGTCCATGCC
*Mcβ-GRP*-F	dsRNA synthesis of β-GRP	CTGGAGGCTTTCGGGTTATTATG
*Mcβ-GRP*-R		TGTTGGTATGAAATGCTCTGGAA
T7-*Mcβ-GRP*-F		ggatcctaatacgactcactataggCTGGAGGCTTTCGGGTTATTATG
T7-*Mcβ-GRP*-R		ggatcctaatacgactcactataggTGTTGGTATGAAATGCTCTGGAA

Nucleotides with lowercase represent the T7 promoter sequence for double-strand RNA synthesis.

### Bioinformatics Analysis

The open reading frame (ORF) of β-GRP was predicted using the ORF Finder program (Stothard 2000). Physical properties, including molecular weight, theoretical isoelectric point (pI), grand average of hydropathicity (GRAVY), protein hydrophobicity (Wilkins et al. 1999), membrane structure (Krogh et al. 2001), signal peptide (Nielsen et al. 2019), phosphorylation site (Blom et al. 2004), and glycosylation site (Gupta et al. 1999), were predicted. Multiple alignment and phylogenetic analysis were performed based on amino acid sequences of recognition protein from *M. cribricollis* (*β-*GRP), *Photinus pyralis* (Linnaeus) (Coleoptera: Lampyridae) (*β-*GRP-like, XP031348780.1), *Agrilus planipennis* Fairmaire (Coleoptera: Buprestidae) (*β-*GRP1-like, XP025830502.1), *Tenebrio molitor* Linnaeus (Coleoptera: Tenebrionidae) (GNBP1, BAG14263.1; *β-*GRP AC99308.1), *Tribolium castaneum* (Herbst) (Coleoptera: Tenebrionidae) (GNBP2, NP001164284.1), *Tribolium madens* (Charpentier) (Coleoptera, Tenebrionidae) (*β-*GRP-like XP044269659.1), *Anopheles gambiae* sensu stricto (Diptera:Culicidae) (GNBP-B1, ABU80032.1), *Nasutitermes comatus* (Isoptera, Nasutitermitinae) (GNBP1, AAZ08480.1), and four glucanases sequences from *Periplaneta americana* (Linnaeus) (Blattodea: Blattidae) (ABR28480.1), *Nocardiopsis sp* (Streptosporangiales: Nocardiopsaceae) (BAE54302.1), *T. molitor* (FJ864682), *Spodoptera frugiperda* (JE Smith) (Lepidoptera : Noctuidae) (ABR28478.2). The catalytic region and catalytic glutamates in glucanases were marked. Multiple alignment of amino acid homologous sequences was carried out using Clustal Omega (Madeira et al. 2019). MEGA 6.0 software was used to construct a phylogenetic tree based on the Neighbor-Joining method with Kimura 2 Parameters model and 1,000 bootstrap replicates.

### Expression Change in *β*-*GRP* under Infection with *M. pingshaense*

In the immune stimulation experiment, healthy *M. cribricollis* larvae were divided into two groups. In one group, *M. cribricollis* were raised in soil treated with *M. pingshaense*. In the other group, *M. cribricollis* were cultured in sterilized soil treated with equal volume of 0.1% Tween 80 (control group). The setting of conidia concentration and soil humidity was described above. Five individuals of *M. cribricollis* larvae were randomly sampled in the experimental and control groups at four time periods (day 0, 7, 12, and 17). Three replicates were set up for each treatment. The tissue was further used for total RNA isolation and subsequent qPCR experiments.

The template cDNA was synthesized for quantitative real-time PCR (qRT-PCR), and the primer pairs for the targeted genes are shown in Table 1. The relative expression levels were determined with BioEasy Master Mix (Bioer, China). qRT-PCR was performed on a Line Gene 9600 Plus real-time fluorescence quantitative PCR instrument (Bioer, China), and data were collected using Gene-9660 software. The qRT-PCT reaction system was according to manufacturer’s instruction. Briefly, 5 μL 2 × SYBR Green Mix, 0.2 μL of each forward and reverse primer (5 μM), 1 μL cDNA template, and 3.6 μL H_2_O in total 10 μL reaction system. The amplification program for qRT-PCR was as follows: 95°C for 2 min, followed by 40 cycles of 95°C for 15 s and 60 °C for 1 min, and a final dissociation cycle of 95°C for 15 s, 60°C for 15 s, and 95°C for 15 s. qRT-PCR was repeated three times for each sample. RPS27a and RPS3 gene was both selected as two housekeeping genes for internal normalization as described in our previous study (Ye et al. 2021), and averaged expression values of targeted genes relative to two housekeeping genes were taken for subsequent analysis. The gene expression level of larvae from control group at day 0 was assumed arbitrarily as 1. The relative mRNA expression level was calculated using the 2^−ΔΔCt^ method (Livak and Schmittgen 2001). Analysis of variance was performed using SPSS software. Statistical significance was analyzed by Student’s *t*-test.

### RNA Interference by Double-strand RNA Injection

The double-strand RNA (dsRNA) of *β*-*GRP* and green fluorescent protein (*GFP*) were synthesized using a cDNA template containing T7 polymerase promoter, and the specific primers are shown in Table 1. dsRNA synthesis was performed using the T7 RiboMAX Express kit (Promega, USA) in accordance with the manufacturer’s instructions. RNA interference was completed via intramuscular injection with 500 ng of dsRNA dissolved in 20 μL of PBS buffer through the connection of the 3rd and 4th abdominal segments of *M. cribricollis* larvae. Larvae of the experimental group were injected with *β*-*GRP* dsRNA, and the control groups were injected with the same amount of PBS buffer or ds*GFP*. At 48 h after injection, five larvae were collected from each group, and the interference effect was detected using qRT-PCR. Larvae treated with PBS group were assumed as control. Three replicates were set up for each treatment. Statistical analysis was performed using SPSS 19.0 software. Significant differences were identified by Student’s *t*-test. Analysis of variance was performed using SPSS software.

### Immune Functional Characterization of *β*-*GRP*

After injecting *β*-*GRP*-specific dsRNA or *GFP*-specific dsRNA or PBS for 48 h, we isolated total RNA from the middle part of the body of *M. cribricollis* and reverse transcribed it to cDNA. The relative expression levels of two AMP genes *defensin* and *lysozyme*, and one *PGRP* gene were analyzed using qRT-PCR, and the primer pairs for the targeted genes are shown in Table 1. Five larvae were randomly sampled in each group with three replicates. Larvae treated with PBS group were assumed as control.

Larvae of three treatment groups injected with PBS, *GFP*, and *β*-*GRP* for 48 h, respectively, then raised in soil treated with *M. pingshaense*. Larvae of three corresponding control groups were raised in soil treated with equal volume of 0.1% Tween 80. The setting of conidia concentration and soil humidity was described above. Each group contained 20 *M. cribricollis*. The experiment was repeated for three times to ensure the accuracy. Thereafter, the number of dead *M. cribricollis* was monitored every 2 d from day 4, and the corrected cumulative mortality was calculated according to the following formula:


$$\begin{array}{l} Corrected\;cumulative\;mortality=\frac{\;\%\;\;test\;mortality-\;\%\;\;control\;mortality}{100-\;\%\;\;control\;mortality}\\ \times 100 \end{array}$$

Analysis of variance was performed using SPSS software. Statistical significance was analyzed by Student’s *t*-test.

## Results

### Molecular Cloning and Bioinformatics Analysis of *β-GRP*

**Fig. 1.**
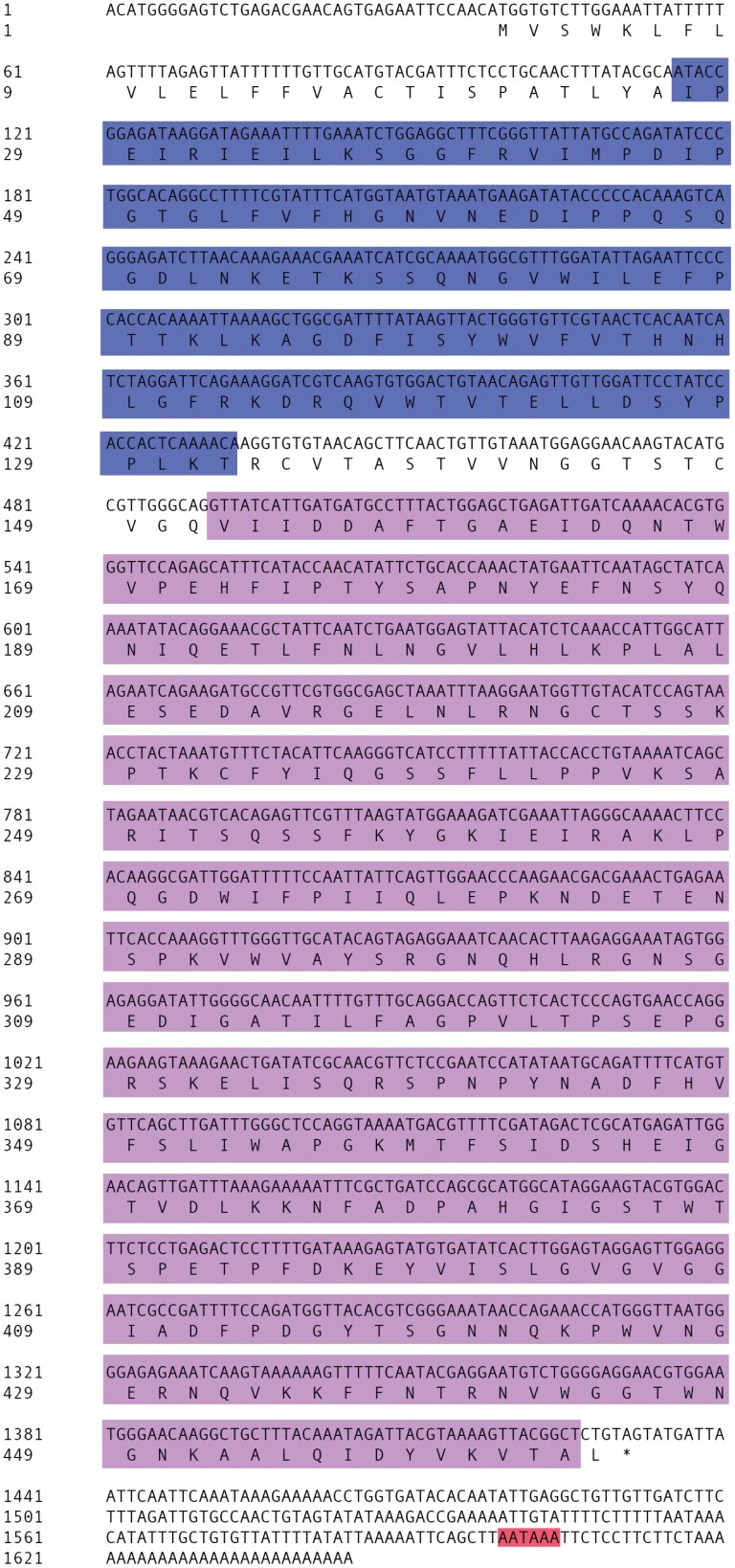
**Sequence of *β-GRP* cDNA nucleotide and translated amino acid.** CBM39 and GH16 binding domain covering residues 27–132 and 152–463, respectively.

The cloned sequence of *β-GRP* cDNA was submitted to the GenBank database (accession NO. MT941530). The full-length was 1644 bp with a complete ORF of 1395 bp encoding a polypeptide consisted of 464 amino acids. The ORF sequence ranged from 38 bp to 1432 bp with starting codon ATG and ending codon TAG. The lengths of 5ʹ UTR and 3ʹ UTR were 37 and 212 bp, respectively (Fig. 1). The putative molecular weight of β-GRP protein was 51.53 kDa, and the pI value was 6.17.

Signal peptide was analyzed. A signal peptide composed of 26 amino acids was found in the N-terminal, and the cleavage site was between positions 26 and 27 (LYA-IP). DictyOGlyc1.1 glycosylation prediction results showed that *β*-GRP had three O-glycosylation sites located at serine sites 325, 330, and 338. TMHMM analysis showed that a typical trans-membrane domain formed between amino acids at positions 7–25, and amino acids at positions 26–464 were located outside the membrane. The *β-*GRP protein was inferred to be hydrophilic based on their few hydrophobic amino acids and many hydrophilic amino acids and GRAVY value of −0.252.

**Fig. 2.**
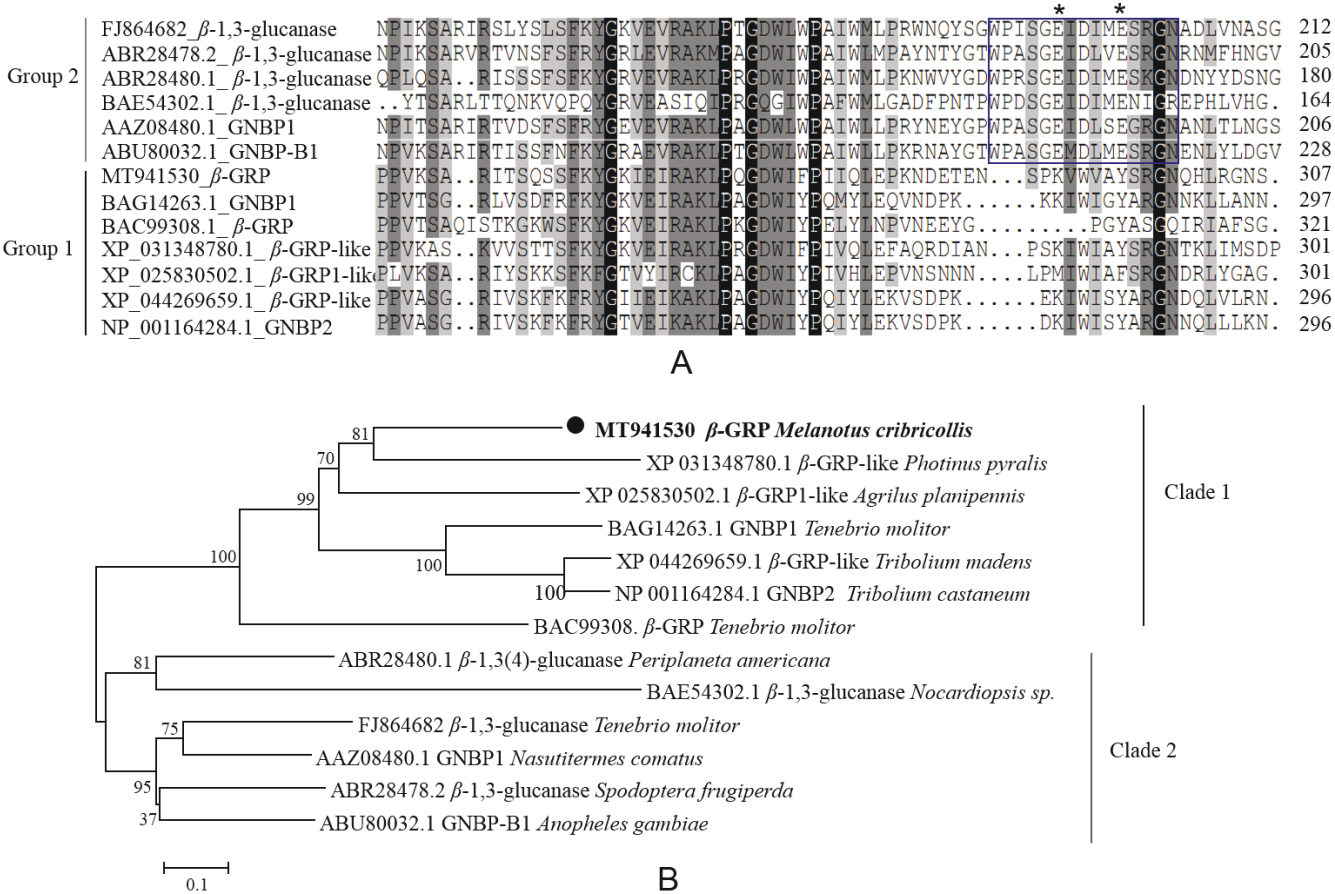
**(A). Multiple alignment of homologous sequences of recognition protein and *β*-1,3-glucanases.** GenBank accession numbers are listed following amino acid sequences. Catalytic region in glucanases is marked with box, and catalytic glutamates are marked with an asterisk. **(B). Phylogenetic tree constructed based on the amino acid sequence of recognition protein and *β*-1,3-glucanases.** The number at the node and the scale bar indicate the bootstrap and the genetic distance, respectively. GenBank accession numbers and species names are also listed.

The results of BlastP showed that the *β*-GRP amino acid sequence contains the β-1,3-glucan recognition protein conserved domain of the insect GRP glycoside hydrolase 16 superfamily (GH16_β-GRP, LamG superfamily) at the C-terminus, and the N-terminus has a carbohydrate binding domain (CBM39 superfamily), covering residues 27–132 and 152–463, respectively (Fig. 1). Sequence comparisons between GH16 domain of several recognition protein sequences and glucanases sequences showed a clear separation. Four glucanases sequences from *P. americana* (ABR28480.1), *Nocardiopsis sp* (BAE54302.1), *T. molitor* (FJ864682), *S. frugiperda* (ABR28478.2), and two recognition protein sequences from *A. gambiae* (GNBP-B1, ABU80032.1) and *N. comatus* (GNBP1, AAZ08480.1) shared a conserved catalytic region with two conserved catalytic glutamates. Except those two recognition protein sequences, no catalytic region was found in other seven recognition protein sequences from *M. cribricollis* (*β-*GRP, MT941530), *P. pyralis* (*β-*GRP-like, XP031348780.1), *A. planipennis* (*β-*GRP1-like, XP025830502.1), *T. molitor* (GNBP1, BAG14263.1; *β-*GRP, AC99308.1), *T. castaneum* (GNBP2, NP001164284.1), *T. madens* (*β-*GRP-like, XP044269659.1) (Fig. 2A). The amino acid sequence of *β-GRP* from *M. cribricollis* clustered with *P. pyralis* into a phylogenetic branch firstly, then clustered into the clade 1 with sequences from *A. planipennis*, *T. molitor*, *T. castaneum*, *T. madens*. Four glucanases sequences cluster into the clade 2 with other two recognition protein sequences from *A. gambiae* and *N. comatus* (Fig. 2B).

### Expression of *β*-*GRP* Stimulates with *M. pingshaense*

**Fig. 3.**
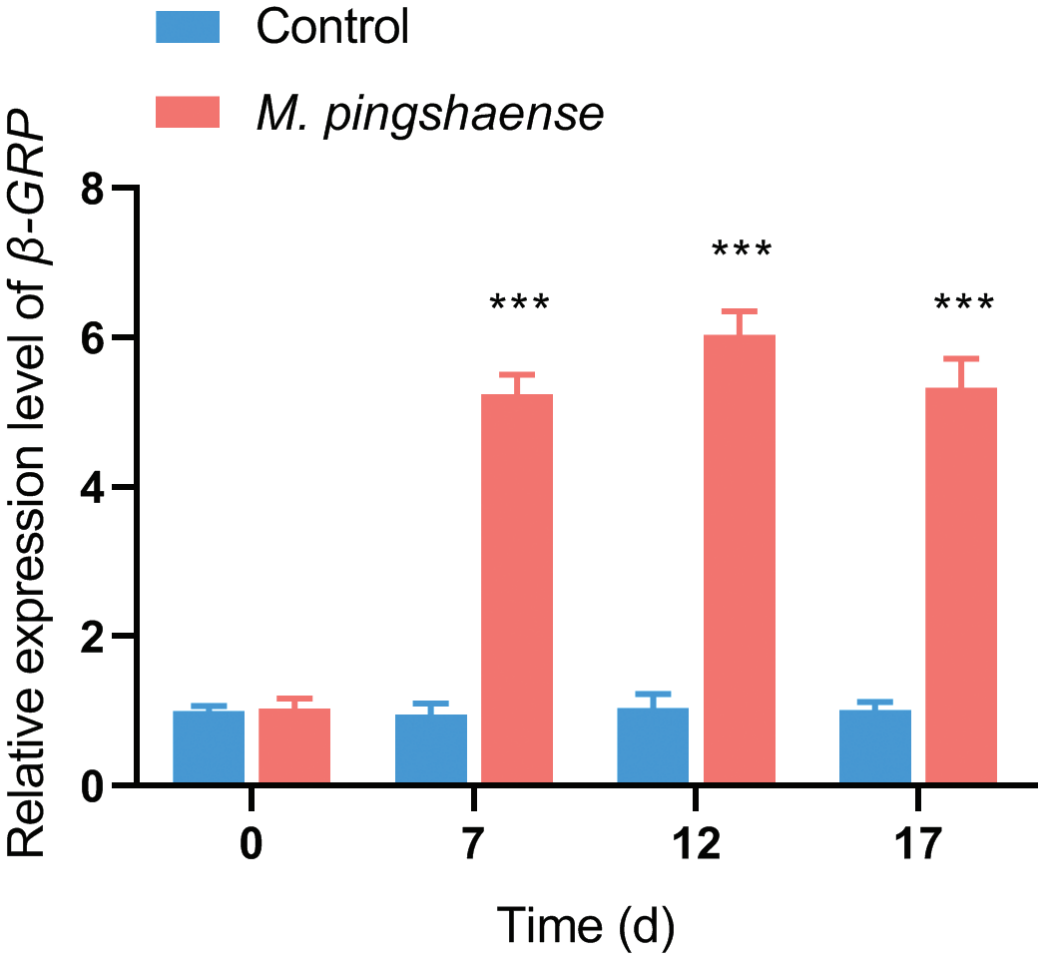
**Changes in the relative transcript expression of *β*-*GRP* after *M. pingshaense* challenge at 0, 7, 12, and 17 d.** Larvae of the treatment group or control group raised in sterilized soil treated with conidia of *M. pingshaense* or equal volume of 0.1% Tween 80, respectively. Five larvae were randomly sampled in each group with three replicates. The gene expression level of larvae from control group at day 0 was assumed arbitrarily as 1. Data are shown as mean ± SEM. Statistical significance was analyzed by Student’s *t*-test (***p < 0.001).

We analyzed the time-course expression profile of *β*-*GRP* to test whether *β-GRP* is involved in the innate immune response of *M. cribricollis* larvae. On day 7, *β*-*GRP* expression significantly upregulated (fold change >5) in the experimental group compared with the control group and remained significantly higher (fold change >5) until day 17 (Fig. 3). This result indicates that *β*-*GRP* probably participated in regulating the immune system of *M. cribricollis* larvae to resist the invasion of pathogenic microorganisms.

### Double Strand RNA Interference

**Fig. 4.**
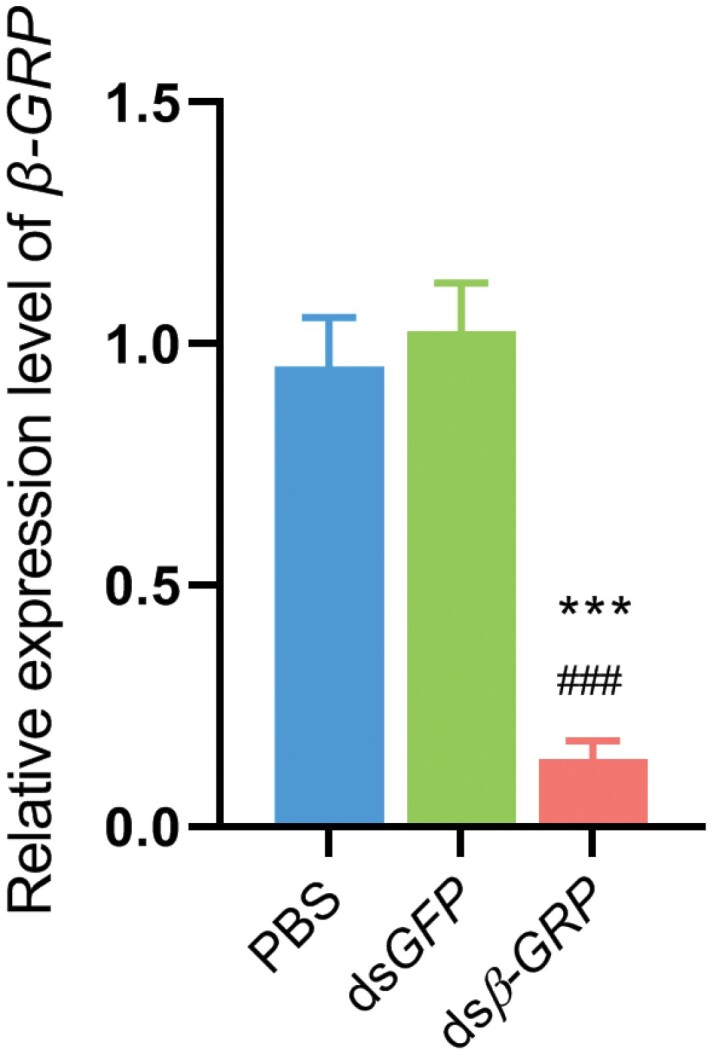
**Efficiency of dsRNA silencing on transcription of *β*-*GRP*.** Each symbol and vertical bar represents the mean ± SEM. Five larvae were randomly sampled in each groups with three replicates. Larvae treated with PBS group were assumed as control. Significant difference between ds*β-GRP* group and PBS group or ds*GFP* group is marked with asterisk (***p < 0.001) or hash symbol (###*p* < 0.001). Statistical significance was analyzed by Student’s *t*-test.

We developed dsRNA interference to confirm the immune function of β-*GRP* in *M. cribricollis* larvae. About 500 bp *β*-*GRP* gene-specific target for dsRNA synthesis were selected. For the negative control group, we synthesized *GFP* gene-specific dsRNA, whose gene does not exist in *M. cribricollis* larvae. As shown in Fig. 4, the expression level of *β*-*GRP* in the *β*-*GRP* interference group was significantly downregulated (fold change >6.5) compared with that in the *GFP* silenced and PBS groups. This result suggested that *β*-*GRP* mRNA was successfully silenced in vivo, and the corresponding dsRNA can be used for further experiment.

### Knockdown of *β*-*GRP* Gene Decreased the Survival Rate with *M. pingshaense* Infection

**Fig. 5.**
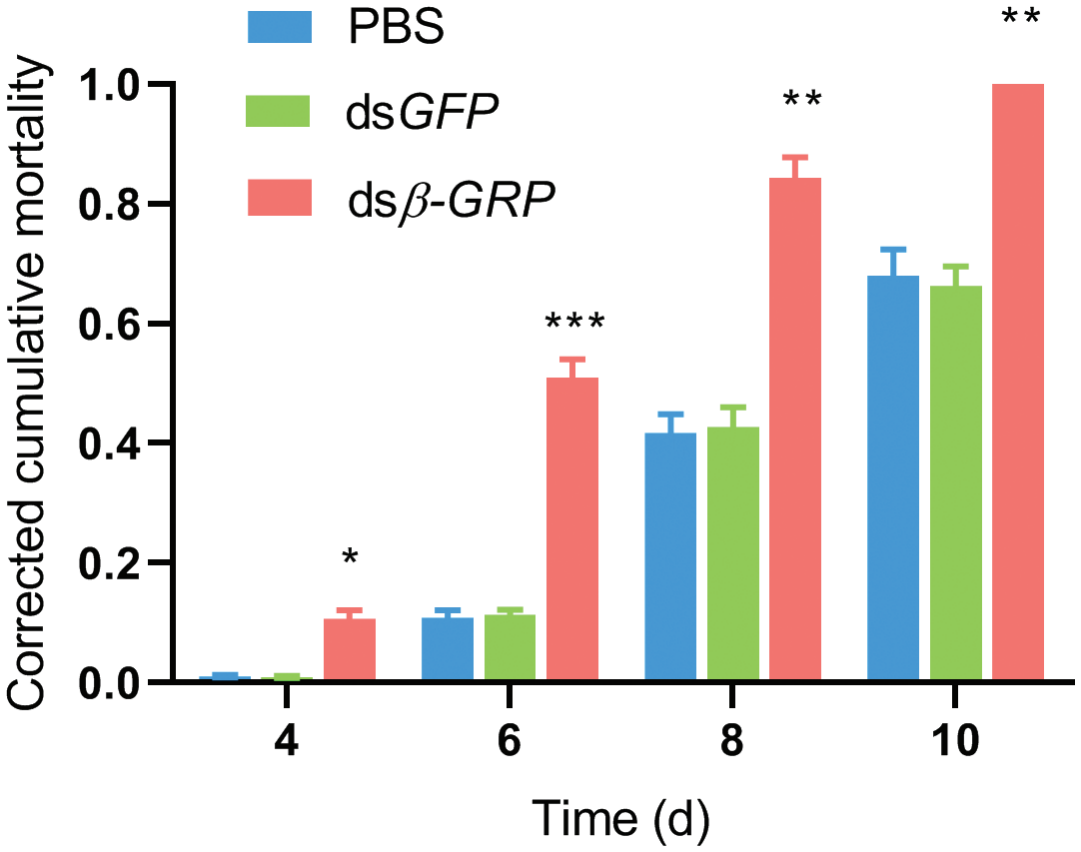
**Survival rates of PBS, ds*GFP*, and ds*β-GRP* treated *M. cribricollis* larvae challenged by *M. pingshaense*.** Mortalities were monitored every 2 d. Larvae of three treatment groups were injected with PBS, *GFP*, and *β-GRP* for 48 h, respectively, then raised in soil treated with *M. pingshaense*. Larvae of three corresponding control groups were raised in soil treated with equal volume of 0.1% Tween 80. Each group contained 20 larvae with three replicates. Each bar represents mean ± SEM. The significant difference between the ds*β-GRP* group and the PBS group or the ds*GFP* group is marked with asterisk (*p < 0.05, **p < 0.01, ***p < 0.001). Data are shown as mean ± SEM. Statistical significance was analyzed by Student’s *t*-test.

**Fig. 6.**
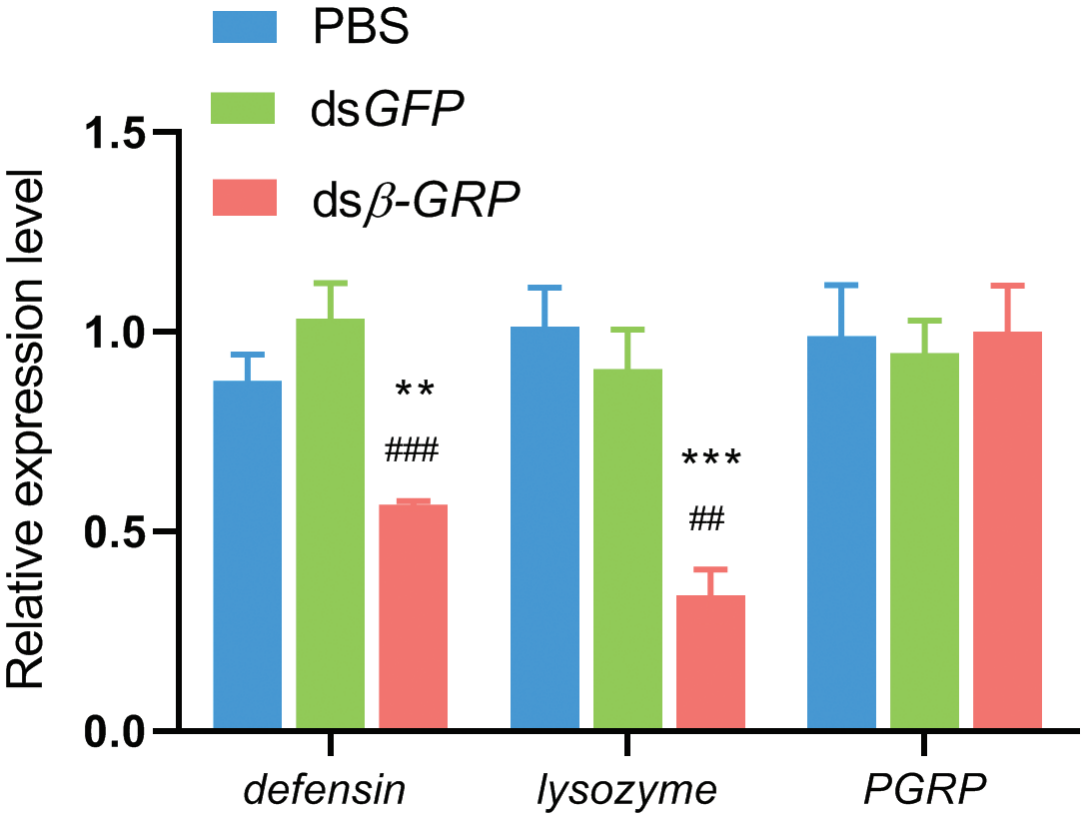
**Expression analysis of antimicrobial peptide gene in *β*-*GRP* silenced *M. cribricollis*.** Each symbol and vertical bar represent mean ± SEM. Five larvae were randomly sampled in each group with three replicates. Larvae treated with PBS group were assumed as control. The significant difference between the ds*β-GRP* group and the PBS group or the ds*GFP* group is marked with asterisk (**p < 0.01, ***p < 0.001) or hash symbol (##*p* < 0.01, ###*p* < 0.001). Data are shown as mean ± SEM. Statistical significance was analyzed by Student’s *t*-test.

The *β*-*GRP* interference group and the control group were further cultured in soil also cultured with *M. pingshaense* to investigate the role of *β*-*GRP* in antifungal immune response. Compared with the control group (ds*GFP* and PBS), the knockdown of *β*-*GRP* significantly decreased survival rate (fold change >10 at day 4) (Fig. 5). This result indicated that *β*-*GRP* can regulate the immune response of *M. cribricollis* larvae to defend against fungal invasion. We also detected the expression level of antimicrobial peptide-related genes after silencing *β*-*GRP* in vivo. As shown in Fig. 6, the expression levels of *defensin* and *lysozyme* in the experimental group were strongly downregulated (fold changes were >2.6 and >1.5, respectively) compared with those in the ds*GFP* and PBS injected groups. This result indicated that *β*-*GRP* probably has the potential to activate the immune system to resist pathogen invasion.

## Discussion

Insect *β-*GRP protein was first identified in *Bombyx mori* and was subsequently discovered in other insects (Dai et al. 2013). Bacterial and fungal cell wall components, including lipopolysaccharide, peptidoglycan, and β-1,3-glucan, are collectively referred to as PAMPs (Janeway 1989). Due to the strong specific affinity for β-1,3-glucan, upon binding with *β-*GRP, it activates the ProPO cascade immediately and then the immune system to prevent bacterial and fungal invasion (Ochiai and Ashida 2000). *β-*GRP is an essential protein to recognize foreign pathogens. However, it’s the molecular characteristics of *β*-GRP and its biological immune function in *M. cribricollis* larvae remain largely unknown.

In the present study, on the basis of the RNA-seq result of *M. cribricollis* larvae we obtained previously, a *β-GRP* homolog gene of *M. cribricollis* larvae was firstly identified, and its immune function was characterized. We first obtained the full-length sequence of *β-GRP* through RACE-PCR. The pI of *β*-GRP protein was 6.17, which was similar to the *β-*GRP pI (6.18) from *Plutella xylostella* ( Linnaeus ) (Lepidoptera: Plutellidae), and both were predicted to be hydrophilic proteins (Chen et al. 2017). β-GRP/GNBP family members have glycoside hydrolase domain of the GH16 superfamily at the C-terminal (Boraston et al. 2004, Cantarel et al. 2009). In this study, *β*-GRP of *M. cribricollis* was predicted to contain conserved GH16 domains (Fig. 1). Insect *β*-1,3-glucanases are similar and evolutionarily related in sequence with recognition protein sequences, which play an important role in pathogen recognition (Genta et al. 2009, Bragatto et al. 2010). In this study, sequence comparisons and phylogenetic analysis results showed *β*-GRP acid sequence was lack of catalytic region, clustered into the same clade with other recognition protein. It was noteworthy that the recognition protein sequences from *A. gambiae* (GNBP-B1, ABU80032.1) and *N. comatus* (GNBP1, AAZ08480.1) shared the conserved catalytic region with glucanases, thus separated with *β*-GRP clade (Fig. 2). Studies have shown that insect *β-*GRPs can be divided into two categories according to the presence or absence of glucanase active site (Panchet et al. 2009).

*β-*GRPs can bind to fungal β-1,3-glucan, activate Toll pathway and ProPO signaling, induce antimicrobial peptide production, and promote melanization cascade (Mishima et al. 2009). The expression of *β-GRP* gene in silkworm epidermis, fat body, and hemolymph increased under fungal infection (Ochiai and Ashida 2000). The expression level of *β-GRP* in *Locusta migratoria manilensis* (Meyen) (Orthoptera: Acrididae) significantly increased after the insects infected by *Metarhizium acridum* (Hypocreales: Clavicipitaceae) (Zheng and Xia 2012). Similarly, after the infection with *M. acridum*, the expression levels of *Tpβ-GRPc* and *Tpβ-GRPd* in the fat body of the *Thitarodes pui* (Lepidoptera, Hepialidae) were significantly upregulated (Sun et al. 2018). Other studies have also confirmed that *β-GRP* is crucial in the immune mechanism of insects against fungal infection (Fabrick et al. 2004, Chen et al. 2017, Zhao et al. 2017). Consistent with the aforementioned results, our study also showed that the time-course profile of *β-GRP* gene from *M. cribricollis* was significantly upregulated after treatment with *M. pingshaense* and maintained a high level (Fig. 4). These results suggested that *β*-*GRP* was probably involved in immune response.

*M. pingshaense* is an effective entomopathogenic fungus of the family Clavicipitaceae that can be applied to pest control (Lovett et al. 2019). In present study, we used *M. pingshaense* as an exogenous pathogen to elucidate how *β-GRP* gene modulates the immune system of *M. cribricollis* larvae under fungal infection. We developed dsRNA interference technique to knockdown the expression level of *β*-*GRP* in vivo, cultured *M. cribricollis* in soil also containing *M. pingshaense*, and then monitored the survival rate of *M. cribricollis*. Compared with that of two groups (ds*GFP* and PBS treated), the mortality of *M. cribricollis* significantly increased. This result indicated that *β-GRP* had the potential to regulate the innate immune system of *M. cribricollis* to defend fungal infection.

Humoral immunity is the dominant barrier to protect the host from pathogen invasion by promoting the release of antimicrobial peptides (Diamond et al. 2009, Sheehan et al. 2018). To demonstrate the molecular mechanism underlying *β-GRP* resistance to pathogen invasion, we evaluated the transcription level of antimicrobial peptides genes *defensin* and *lysozyme* after *β-GRP* interference. After silencing *β-GRP* in vivo, the expression of *defensin* and *lysozyme* significantly declined. This result indicates that *β-GRP* might regulate the body’s antifungal immune response by inducing the expression of antimicrobial peptides.

In summary, a novel *β-*GRP of *M. cribricollis* was first characterized. The results suggest that this gene probably plays a crucial role to stimulate the expression of antimicrobial peptides in *M. cribricollis* larva against fungal infection. Our findings would enrich the understanding of *β-GRP* function in the innate defense immune mechanism of insects.
